# Improved outcomes of localized diffuse large B‐cell lymphoma at the Waldeyer ring in comparison to the sinonasal area in the rituximab era

**DOI:** 10.1002/cam4.6851

**Published:** 2023-12-26

**Authors:** Wei‐Li Ma, Ruey‐long Hong, Pei‐Jen Lou, Ming Yao, Shang‐Ju Wu, Chung‐Wu Lin, Chun‐Wei Wang, Chin‐Hao Chang, Ann‐Lii Cheng, Sung‐Hsin Kuo

**Affiliations:** ^1^ Department of Oncology National Taiwan University Hospital and National Taiwan University College of Medicine Taipei City Taiwan; ^2^ Cancer Research Center National Taiwan University College of Medicine Taipei City Taiwan; ^3^ Graduate Institute of Oncology National Taiwan University College of Medicine Taipei City Taiwan; ^4^ Department of Medical Oncology National Taiwan University Cancer Center, National Taiwan University College of Medicine Taipei City Taiwan; ^5^ Department of Otolaryngology National Taiwan University Hospital and National Taiwan University College of Medicine Taipei City Taiwan; ^6^ Department of Internal Medicine National Taiwan University Hospital and National Taiwan University College of Medicine Taipei City Taiwan; ^7^ Department of Pathology National Taiwan University Hospital and National Taiwan University College of Medicine Taipei City Taiwan; ^8^ Department of Pathology and Laboratory Medicine Koo Foundation Sun Yat‐Sen Cancer Center Taipei City Taiwan; ^9^ Department of Medical Research National Taiwan University Hospital and National Taiwan University College of Medicine Taipei City Taiwan; ^10^ Department of Radiation Oncology National Taiwan University Cancer Center, National Taiwan University College of Medicine Taipei City Taiwan

**Keywords:** DLBCL, extranodal, prognosis, R‐IPI score, sinonasal area, Taiwan, Waldeyer ring

## Abstract

**Background:**

Diffuse large B‐cell lymphoma (DLBCL) of the head‐and‐neck area primarily involves the Waldeyer ring (WR) and sinonasal area (SN). However, the differential clinical outcomes between patients with WR‐DLBCL and those with SN‐DLBCL in the rituximab era remain unclear.

**Methods:**

To avoid confounding factors contributed by advanced DLBCL with WR and SN involvement, we assessed the clinical outcomes of patients with stage I/II WR‐DLBCL and SN‐DLBCL and compared them with those having corresponding stages of DLBCL in the lymph nodes but without other extranodal involvement (LN‐DLBCL) in the same period. We compared the patients' clinical characteristics, treatment modalities, event‐free survival (EFS), and overall survival (OS) among the three subgroups.

**Results:**

We analyzed 67, 15, and 106 patients with WR‐DLBCL, SN‐DLBCL, and LN‐DLBCL, respectively, between January 2000 and December 2019. All patients received front‐line rituximab‐based regimens, and > 80% received rituximab, cyclophosphamide, doxorubicin, vincristine, and prednisolone‐based regimens. More patients with SN‐DLBCL had revised International Prognostic Index (R‐IPI) score 3 (27%) when compared with those with WR‐DLBCL (7%) and those with LN‐DLBCL (10%, *p* = 0.181). Patients with WR‐DLBCL, LN‐DLBCL, and SN‐DLBCL had 5‐year EFS and OS rates of 80.7%, 59.5%, and 41.9% (*p* = 0.021) and 83.7%, 70.8%, and 55.8% (*p* = 0.032), respectively. Compared to patients with LN‐DLBCL, those with WR‐DLBCL also had a significantly favorable 5‐year EFS rate (*p* = 0.021) and 5‐year OS rate (*p* = 0.023). Three of the 15 patients with SN‐DLBCL experienced lymphoma recurrence in the brain after front‐line treatment. In multivariate analyses, R‐IPI scores of 1–2 and 3 served as significantly poor prognostic factors for patients with poor EFS and OS.

**Conclusions:**

Compared to patients with LN‐DLBCL, patients with WR‐DLBCL receiving front‐line rituximab‐based treatments had favorable clinical outcomes; however, patients with SN‐DLBCL had worse clinical outcomes. Further studies on molecular prognostic factors and treatment strategies for SN‐DLBCL are warranted.

## Graphical abstract

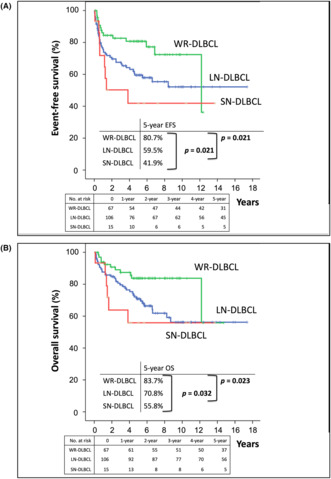

## 1 INTRODUCTION

Diffuse large B‐cell lymphoma (DLBCL) is the most common subtype of aggressive non‐Hodgkin lymphoma (NHL) worldwide, occurring in 30% of Caucasians with lymphomas and up to 40% of all lymphomas in Asians.^1^ Two studies showed a higher incidence of DLBCL in Taiwan where it accounted for 48% and 44% of total lymphomas, respectively.^2^, ^3^ Previous randomized trials have demonstrated that patients receiving front‐line rituximab (R)‐based immunochemotherapy, that is, cyclophosphamide, doxorubicin hydrochloride, vincristine sulfate, and prednisolone (R‐CHOP), have better clinical outcomes, including even‐free survival (EFS) and overall survival (OS) rates compared to those receiving CHOP, especially in older (aged 60–80 years), or young (aged 18–60 years) patients with good clinical prognosis.^4^, ^5^ The International Prognostic Index (IPI) scoring^6^ is calculated by the following items: age, stages, performance status of Eastern Cooperative Oncology Group (ECOG), serum lactate dehydrogenase (LDH) level, and engagements of extranodal sites. Compared with the standard IPI score, the revised IPI (R‐IPI) score can offer predictive value for clinical outcomes (progression‐free survival [PFS] and OS) in patients with DLBCL after receiving R‐CHOP‐based regimens.^7^

DLBCL arising from tissues other than lymph nodes is called extranodal DLBCL, of which primary central nervous system (CNS) lymphoma and primary cutaneous DLBCL, leg type are the most well‐known subtypes.^8^ In addition to histology of squamous cell carcinoma and salivary gland neoplasm, NHL, the third most common malignancy, occurs in the oral cavity and maxillofacial region, in which Waldeyer ring (WR), including lingual, palatine, pharyngeal, and tubal tonsils, is the most common site.^9^, ^10^ In addition to histology subtype of mucosa‐associated lymphoid tissue, the most common subtype of lymphomas involving WR is DLBCL.^9^, ^10^ DLBCL also involves the other head‐and‐neck area, including the nasal cavity and paranasal sinus, termed as sinonasal area (SN).^11^

In the pre‐rituximab era, patients with DLBCL located in the WR (WR‐DLBCL) or DLBCL located in the SN (SN‐DLBCL) mostly received CHOP‐based regimens followed by or without consolidative radiotherapy (RT) to the involved field of the WR or SN.^12^, ^13^, ^14^, ^15^, ^16^, ^17^, ^18^ In the aforementioned reports,^12^, ^13^, ^14^, ^15^, ^16^, ^17^, ^18^ when comparing with patients with nodal DLBCL, those with WR‐DLBCL had better or similar outcomes. However, comparisons of clinical outcomes between patients with SN‐DLBCL and nodal DLBCL have rarely been reported.^12^, ^13^, ^14^, ^15^, ^16^, ^17^, ^18^

Based on most reports of patients with head‐and‐neck DLBCL treated with CHOP‐based regimens and studies that included nodal DLBCL with possible metastasis to the WR and SN,^19^ we assessed the clinicopathological features, R‐IPI scores, immunochemotherapy regimens with or without RT, and EFS and OS of patients with localized (stage I/II) WR‐DLBCL or SN‐DLBCL who received R‐CHOP‐based regimens in the current study. Furthermore, we compared WR‐DLBCL and SN‐DLBCL patient characteristics, EFS to front‐line treatments, and OS with those diagnosed with DLBCL involving the lymph nodes but without any other extranodal sites (LN‐DLBCL) in the same period. Finally, we assessed the prognostic factors affecting the EFS and OS of patients with localized head‐and‐neck DLBCL in the rituximab era. We also assessed the expression patterns of MYC, BCL2, p53, and CD5 in the available tissue samples from the same group of patients.

## 2 MATERIALS AND METHODS

### 2.1 Patient selection, stratification, and data analysis

**FIGURE 1:**
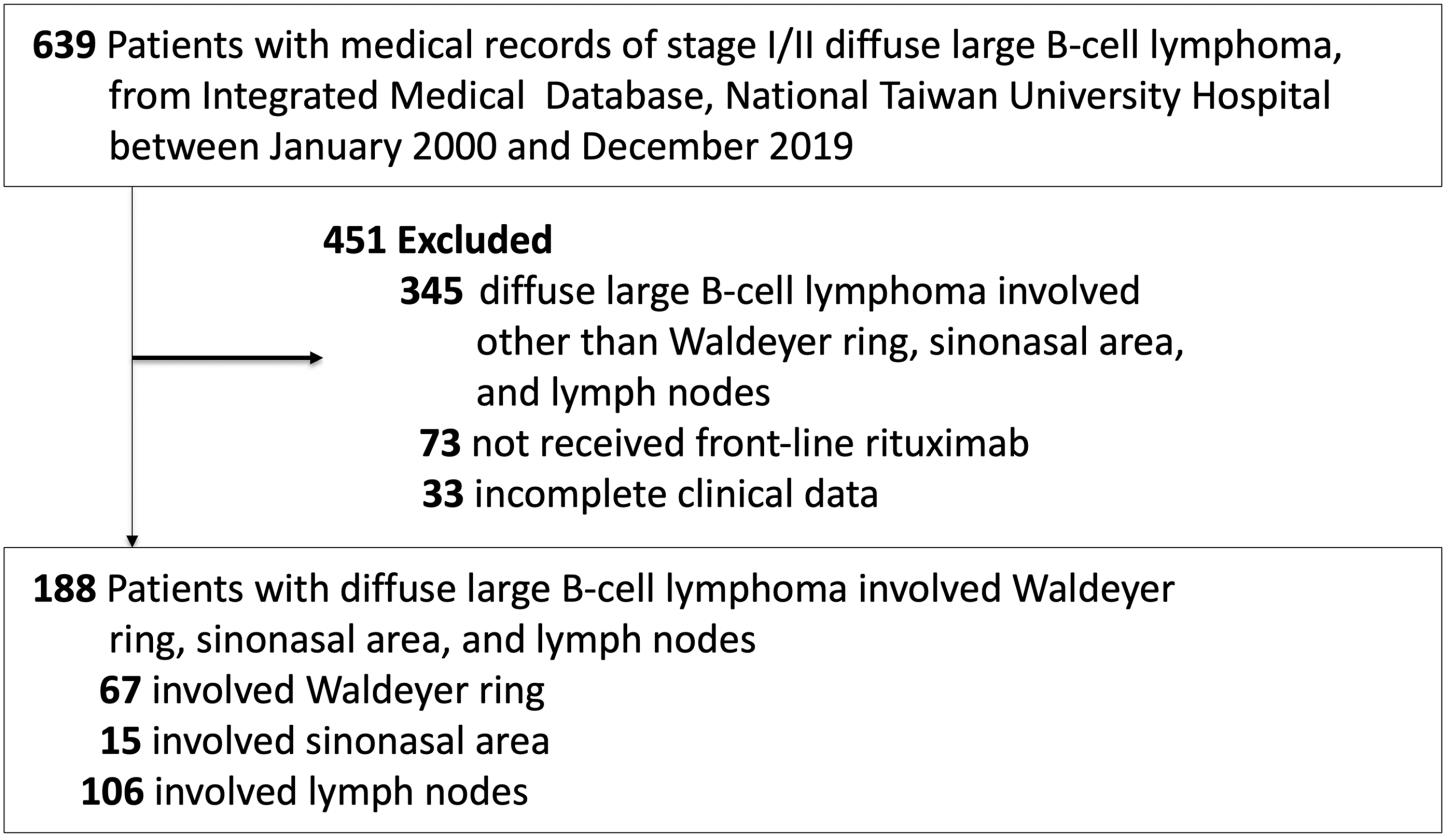
The CONSORT diagram showing selection and disposition of patients with diffuse large B‐cell lymphoma involving the Waldeyer ring (WR‐DLBCL), sinonasal area (SN‐DLBCL), and lymph nodes without extranodal involvement (LN‐DLBCL).

In the present study, we included patients with stage I/II WR‐DLBCL whose DLBCL involved the lingual, palatine, pharyngeal, or tubal tonsils and those with stage I/II SN‐DLBCL whose DLBCL involved the nasal cavity or paranasal sinuses. During the same period, we included patients with stage I/II LN‐DLBCL (control group), and their outcomes were compared with those of patients with WR‐DLBCL and SN‐DLBCL. Because DLBCL in advanced stages may concurrently involve multiple lymph nodes and organs, including the WR and SN, we only studied stage I/II WR‐DLBCL and SN‐DLBCL and compared the corresponding stages of LN‐DLBCL for analysis. We defined patients as having stage I WR‐DLBCL or SN‐DLBCL involving only the WR or SN and stage II WR‐DLBCL or SN‐DLBCL involving other lymph nodes above the diaphragm. We retrieved the medical records of clinical data of patients with stage I/II DLBCL diagnosed and treated between January 2000 and December 2019 from the Integrated Medical Database (IMD) of our institute, National Taiwan University Hospital (NTUH). Based on the IMD of NTUH, we excluded 451 patients from the potentially 639 eligible patients, including 345 whose DLBCL involved sites other than the WR, SN, or lymph nodes, 73 who did not receive frontline rituximab, and 33 who had inadequate medical information. Finally, we analyzed the clinicopathological features and outcomes of 188 patients, including 67 with WR‐DLBCL, 15 with SN‐DLBCL, and 106 with LN‐DLBCL (Figure 1).

The pathologist confirmed that the diagnosis of DLBCL based on the revised criteria of the lymphoid neoplasms of the 2016 World Health Organization classification.^8^ Patients' age, sex, lymphoma involvement areas, ECOG performance status, blood cell counts, serum chemistry study (including LDH and β2‐microglobulin), viral hepatitis, human immunodeficiency virus (HIV) status, and bone marrow study at diagnosis were retrieved from the medical records. Among the 188 patients, most patients (145 [77%]) underwent positron emission tomography‐computed tomography (PET‐CT) and 43 (23%) underwent whole‐body CT examinations at initial diagnosis. The clinical stages of DLBCL were determined using the Lugano classification.^20^ The standard IPI scores of the patients at diagnosis were calculated according to >60 years of age, III or IV of Ann Arbor stage, ECOG performance status ≥2, serum LDH level >1 normal range, and >1 extranodal site.^6^ However, in the current study, our patients were all stage I/II, had no more than one extranodal site, and all received front‐line rituximab‐based treatment; thus, we used R‐IPI scores (> 60 years of age, ECOG performance status ≥2, and serum LDH level >1 normal range),^7^ including 0 (very good), 1–2 score (good), and 3 (poor) to predict patient prognosis. In the rituximab era, bulky DLBCL was defined as that with a diameter of >6–10 cm; in this study, we defined bulky DLBCL as a DLBCL with a diameter of >10 cm.^4^, ^20^ All patients received 4–6 cycles of front‐line regimens where the dosage and number of cycles for each patient was determined by their attending physician.^4^, ^5^ In 2011, R‐miniCHOP (rituximab IV [intravenous] 375 mg/m^2^, cyclophosphamide IV 400 mg/m^2^, doxorubicin IV 25 mg/m^2^, vincristine IV 1 mg on day 1; oral prednisone 40 mg/m^2^ on days 1–5) was demonstrated to provide optimal efficacies (2‐year PFS, 47%; OS, 59%) and safety (39.3% cases developed grade ≥3 neutropenia) for patients with DLBCL and over the age of 80 years old, through a multicenter, single‐arm, phase 2 trial.^21^ Elderly patients (age > 80 years) with newly diagnosed DLBCL at our institute started treatment with the front‐line R‐miniCHOP regimen.

In our institute, patients with SN‐DLBCL who received prophylactic intra‐thecal chemotherapy were dependent on the decisions of clinical physicians according to the R‐IPI score, comorbidities, age, and higher risk of CNS recurrence. The intra‐thecal chemotherapy regimens in our study contained 15 mg of methotrexate, 40 mg of cytarabine, and 50 mg of hydrocortisone, and were administered during every systemic front‐line treatment for at least 3–4 cycles. Patients would receive consolidative RT after front‐line systemic treatment with an RT dose of at least 30 Gy.^22^ Patients developing relapsed or refractory disease after front‐line systemic treatments would be treated with autologous stem cell transplantation when having feasible clinical conditions.^23^

After initiating frontline treatments, all patients underwent whole‐body CT scans every 3 months, according to their clinical conditions or clinical judgment. Some patients also underwent additional CT scans between the 3‐month interval and PET‐CT to evaluate the disease status at the discretion of their clinical physicians. We calculated EFS as the time from front‐line treatment until treatment failure, including recurrent or progressive disease, or patient death. We defined OS as the interval from the time of front‐line treatment to the time of bereavement from any cause of lymphoma.

The requirement for informed consent was waived because all patient identifiers were removed from the dataset prior to data analysis. This study was conducted in accordance with the principles of the Declaration of Helsinki and its amendments. This retrospective clinical cohort study and this translational study were approved by the Research Ethics Committee (REC) of the NTUH (approval number: NTUHREC No. 201912008RINA; and No. 202103051RINB).

### 2.2 Immunohistochemical (IHC) staining and scoring

Furthermore, we assessed the protein expression patterns of MYC, BCL2, p53, and CD5 by IHC staining in patients with available paraffin‐embedded pathology tissue samples at the time of diagnosis, to investigate whether these molecules could predict the prognosis of these patients. Paraffin‐embedded sections (5 μm thick) were deparaffinized and rehydrated using xylene and ethanol. Antihuman primary antibodies against MYC (clone Y69; ab32072; Abcam, Cambridge, UK), BCL2 (clone SP66; ab236221; Abcam), p53 (clone DO7; 453 M‐96; Cell Marque, Rocklin, California, United States), and CD5 (clone SP19; 205R‐16; Cell Marque) were used to detect the corresponding proteins. These slides were incubated with biotin‐conjugated secondary antibodies, and antibody binding was detected using avidin‐biotin enzyme reagent. In this study, MYC, BCL2, p53, and CD5 were independently scored by expert pathologists who were blinded to the clinical data. Expression of MYC and BCL2 was considered positive if MYC proteins (indicated by nuclear staining) were detected in >40% of lymphoma cells and BCL2 proteins (indicated by membranous staining) were detected in >50% of lymphoma cells.^24^ Expression of p53 was defined as positive nuclear staining of p53 in >50% of lymphoma cells, whereas expression of CD5 was defined as positive membranous staining of CD5 in >25% of lymphoma cells.^25^, ^26^

### 2.3 Statistical analysis

We compared the distributions of categorical variables using Fisher's exact test and continuous variables and medians using the Kruskal–Wallis test in all patients. In addition, we calculated the patients' EFS and OS using the Kaplan–Meier method. Finally, we used univariate and multivariate Cox proportional hazards models to explore the associations between EFS or OS and the following clinical characteristics: patient age, sex, ECOG performance status, LDH, β2‐microglobulin, stage I or II disease, R‐IPI scores, and DLBCL subgroups according to the involvement sites: WR‐, SN‐, and LN‐DLBCL. The *p* values <0.05 indicated significance.

## 3 RESULTS

### 3.1 Characteristics and treatment modalities of patients

The clinical characteristics and treatment modalities of the 188 patients with WR‐DLBCL, SN‐DLBCL, and LN‐DLBCL are listed in Table 1. Among the 67 patients with WR‐DLBCL, 12 (18%), 42 (63%), and 13 (19%) had DLBCL in the lingual tonsil, palatine tonsil, and pharyngeal and tubal tonsils, respectively. Among the 15 patients with SN‐DLBCL, eight (53%) had DLBCL in the nasal cavity and seven (47%) had DLBCL in the paranasal sinus. Patients' median ages were 64, 73, and 65 years for WR‐DLBCL, SN‐DLBCL, and LN‐DLBCL, respectively (*p* = 0.167). There were more patients aged >60 years in SN‐DLBCL (*n* = 13, 87%) than those with WR‐DLBCL (*n* = 42, 63%) and LN‐DLBCL (*n* = 61, 58%). However, the differences among the three groups were not statistically significant (*p* = 0.093). Patients with SL‐DLBCL (47%) had a trend of worsening ECOG performance status (≥2) than those with WR‐DLBCL (25%) and with LN‐DLBCL (25%) (*p* = 0.185). Furthermore, patients with WR‐DLBCL had a higher probability of a normal LDH level (73%) than that of patients with SN‐DLBCL (47%) and of patients with LN‐DLBCL (51%) (*p* = 0.010). Similarly, patients with WR‐DLBCL had a lower probability of an abnormal β2‐microglobulin level (10%) when compared with those with SL‐DLBCL (20%) and with LN‐DLBCL (26%) (*p =* 0.039). However, there was no significant difference in B symptoms among the three subgroups of our patients (*p* = 0.491, Table 1). Stage I was found in 31% of WR‐DLBCL cases and 24% of LN‐DLBCL cases, whereas stage II was found in 69% of WR‐DLBCL cases and 76% of LN‐DLBCL cases, respectively. Patients with SN‐DLBCL had more stage II (87%) than stage I (13%) tumors; however, the difference in the distribution of stages between WR‐DLBCL, SN‐DLNCL, and LN‐DLBCL was not significant (*p* = 0.276).

**TABLE 1:** Characteristics of patients with diffuse large B‐cell lymphoma involving the Waldeyer ring, sinonasal area, or lymph nodes without extranodal involvement.

	DLBCL in the head‐and‐neck area		Comparison cohort	*p* Value
	WR‐DLBCL (*n* = 67)	SN‐DLBCL (*n* = 15)	LN‐DLBCL (*n* = 106)	
Locations at the head‐and‐neck area, *n* (%)				
Lingual tonsil	12 (18%)			
Palatine tonsil	42 (63%)			
Pharyngeal and tubal tonsil	13 (19%)			
Nasal cavity		8 (53%)		
Paranasal sinus		7 (47%)		
Age (years)				
Median (range)	64 (34–93)	73 (45–91)	65 (21–92)	0.167
> 60, *n* (%)	42 (63%)	13 (87%)	61 (58%)	0.093
Sex, *n* (%)				0.515
Male	34 (51%)	10 (67%)	59 (56%)	
Female	33 (49%)	5 (33%)	47 (44%)	
ECOG performance status, *n* (%)				0.185
0–1	50 (75%)	8 (53%)	80 (75%)	
≥ 2	17 (25%)	7 (47%)	26 (25%)	
Serum LDH level, *n* (%)				0.010
> 1 normal range	18 (27%)	8 (53%)	52 (49%)	
Serum β2‐microglobulin, *n* (%)				0.039
> upper limit of normal range	7 (10%)	3 (20%)	28 (26%)	
B symptoms, *n* (%)	11 (16%)	2 (13%)	24 (23%)	0.491
Stages, *n* (%)				0.276
Stage I	21 (31%)	2 (13%)	25 (24%)	
Stage II	46 (69%)	13 (87%)	81 (76%)	
R‐IPI scores, *n* (%)				0.181
0	17 (25%)	1 (7%)	26 (25%)	
1–2	45 (67%)	10 (67%)	69 (65%)	
3	5 (7%)	4 (27%)	11 (10%)	
Bulky disease (>10 cm in diameter), *n* (%)	1 (1%)	1 (7%)	7 (7%)	0.289
HIV infection, n (%)				0.458
Anti‐HIV (+)	0 (0%)	0 (0%)	2 (2%)	
Viral hepatitis, n (%)				
HBsAg (+)	7 (10%)	0 (0%)	21 (20%)	0.058
Anti‐HCV (+)	3 (4%)	0 (0%)	4 (4%)	0.709
Front‐line immunochemotherapy regimens, *n* (%)				0.291
Rituximab (+), anthracycline (+); (R‐CHOP‐based regimens)	59 (88%)	14 (93%)	86 (81%)	
Rituximab (+), anthracycline (−)	8 (12%)	1 (7%)	20 (19%)	
Consolidative radiotherapy, *n* (%)				0.013
Received radiotherapy	9 (13%)	7 (47%)	20 (19%)	
Not received radiotherapy	58 (87%)	8 (53%)	86 (81%)	
Autologous stem‐cell transplantation after failed front‐line treatments, *n* (%)	3 (4%)	1 (7%)	8 (8%)	0.723

Abbreviations: anti‐HCV, hepatitis C virus antibody; anti‐HIV, human immunodeficiency virus antibody; ECOG, Eastern Cooperative Oncology Group; HBsAg, hepatitis B virus surface antigen; LN‐DLBCL, diffuse large B‐cell lymphoma involving lymph nodes without extranodal involvement; LDH: lactate dehydrogenase; R‐IPI scores, revised International Prognostic Index scores; R‐CHOP, rituximab, cyclophosphamide, doxorubicin, vincristine, and prednisolone; SN‐DLBCL, diffuse large B‐cell lymphoma involving the sinonasal area; WR‐DLBCL, diffuse large B‐cell lymphoma involving the Waldeyer ring.

More patients with SN‐DLBCL had R‐IPI score of 3: five (7%) patients with WR‐DLBCL, four (27%) patients with SN‐DLBCL, and 11 (10%) patients with LN‐DLBCL had R‐IPI score of 3 (*p* = 0.181). Only one (1%) patient with WR‐DLBCL, one (7%) with SN‐DLBCL, and eight (7%) with LN‐DLBCL had bulky disease (>10 cm in diameter), and two (2%) patients with LN‐DLBCL had concurrent HIV infection.

Seven (10%) and 21 (20%) patients with WR‐DLBCL and LN‐DLBCL, respectively, were positive for serum hepatitis B antigen and received prophylactic antiviral drugs or were followed up by hepatologists at our institute during systemic treatments. Ultimately, 59 patients (88%) with WR‐DLBCL, 14 (93%) with SN‐DLBCL, and 86 (81%) with LN‐DLBCL received front‐line immunochemotherapy with rituximab and anthracycline (R‐CHOP‐based regimens). Eight patients (12%) with WR‐DLBCL, one (7%) with SN‐DLBCL, and 20 (19%) with LN‐DLBCL received front‐line rituximab‐based regimens but without anthracycline. The number of patients with SN‐DLBCL who received consolidative RT (*n* = 7, 47%) was higher than that of those with WR‐DLBCL (*n* = 9, 13%) or LN‐DLBCL (*n* = 20, 19%; *p* = 0.013). Three (4%), one (7%), and eight (8%) patients with WR‐DLBCL, SN‐DLBCL, and LN‐DLBCL, respectively, underwent autologous stem cell transplantation after failing front‐line treatments (*p* = 0.723).

Among our patients, 36 patients were over the age of 80 years, including 18 LN‐DLBCL, 14 WR‐DLBCL, and 4 SN‐DLBCL (Table 2). Among these, four patients received standard R‐CHOP, eight patients received reduced‐dose (80% of anthracycline) of R‐CHOP, and 9 patients received R‐miniCHOP. The remaining 15 patients received rituximab‐based therapy with no anthracycline‐based, front‐line regimens. Among 12 patients receiving a standard‐ or reducing‐ dose of R‐CHOP, four patients were diagnosed before 2011 and eight patients were diagnosed after 2011; and all patients presented with no comorbidities at initial diagnosis.

**TABLE 2:** Clinical information of 15 patients with diffuse large B‐cell lymphoma involving the sinonasal area after front‐line rituximab‐based regimens.

No.	Sex	Age	ECOG performance status	LDH >1 normal range	β2‐microglobulin> upper limit of normal range	B symptoms	Stage	R‐IPI scores	Front‐line chemotherapy	Front‐line radiotherapy	CNS prophylaxis	Disease relapse after front‐line chemotherapy	CNS relapse after front‐line chemotherapy	Status	Follow‐up (ys)
1	F	70	2	N	N	N	2	2	R‐CHOP	N	Y	Y	Y (HDMTX)	Dead	0.1
2	M	74	2	N	N	N	2	2	R‐CHOP	N	Y	Y	Y (R‐HDMTX/HDAC, R‐EHDAC)	Alive	4.2
3	M	67	0	Y	Y	N	2	2	R‐CHOP	N	N	Y (ICE)	N	Alive	3.9
4	M	61	0	N	N	N	2	1	R‐CHOP	N	N	Y (R‐ESHAP+ASCT)	N	Dead	1.5
5	M	91	3	Y	Y	N	1	3	R‐COP	Y	N	N	N	Alive	0.6
6	M	58	0	N	N	N	1	0	R‐CHOP	N	N	Y	Y (R‐BAS, WBRT)	Dead	1.6
7	F	83	2	Y	N	N	2	3	R‐CHOP	Y	Y	N	N	Alive	2.8
8	M	73	1	Y	Y	N	2	2	R‐CHOP	N	N	N	N	Alive	5.7
9	F	62	0	N	N	N	2	1	R‐CHOP	Y	N	N	N	Alive	10.9
10	F	45	0	Y	N	N	2	1	R‐CHOP	N	N	N	N	Alive	13.7
11	F	65	0	N	N	N	2	1	R‐CHOP	N	N	N	N	Alive	10.4
12	M	88	2	Y	N	Y	2	3	R‐miniCHOP	Y	N	N	N	Dead	3.8
13	M	84	2	N	N	N	2	2	R‐miniCHOP	Y	N	N	N	Dead	1.2
14	M	76	2	Y	N	N	2	3	R‐CHOP	Y	N	N	N	Alive	5.0
15	M	74	1	Y	N	Y	2	2	R‐CHOP	Y	N	N	N	Dead	1.4

Abbreviations: ASCT, autologous stem cell transplantation; CNS, central nervous system; ECOG, Eastern Cooperative Oncology Group; ICE, ifosfamide, carboplatin, and etoposide; F, female; HDMTX, high‐dose methotrexate; LDH, lactate dehydrogenase; M, male; N, yes; No, number; R‐IPI scores, revised International Prognostic Index scores; R‐CHOP, rituximab, cyclophosphamide, doxorubicin, vincristine, and prednisolone; R‐miniCHOP, rituximab and reduced‐dose CHOP; R‐COP, rituximab, cyclophosphamide, vincristine, and prednisolone; R‐ESHAP, rituximab, etoposide, methylprednisolone, cytarabine, and cisplatin; R‐HDMTX/HDAC, rituximab, high‐dose methotrexate and high dose cytarabine; R‐EHDAC, rituximab, etoposide and high‐dose cytarabine; R‐BAS, rituximab, carmustine (BCNU), high‐dose cytarabine and steroid; ys, years; WBRT, whole‐brain radiotherapy.

### 3.2 Clinical outcomes and prognostic factors of patients with WR‐DLBCL, SN‐DLBCL, and LN‐DLBCL

**FIGURE 2:**
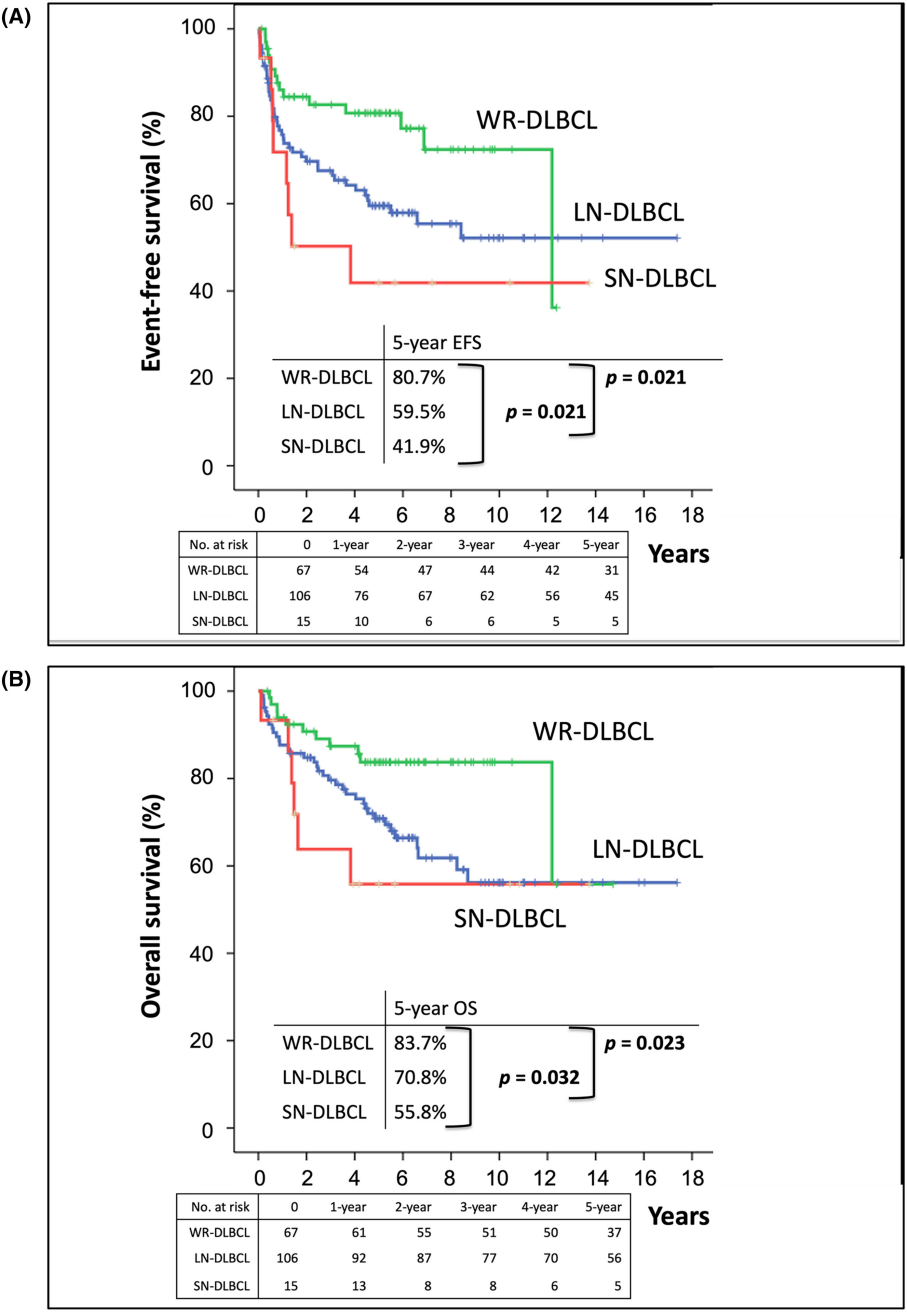
Differences in event‐free survival (EFS) and overall survival (OS) in patients with diffuse large B‐cell lymphoma involving the Waldeyer ring (WR‐DLBCL), sinonasal area (SN‐DLBCL), and lymph nodes without extranodal involvement (LN‐DLBCL) (A) The 5‐year EFS rates of patients with WR‐DLBCL, LN‐DLBCL, and SN‐DLBCL were 80.7%, 59.5%, and 41.9%, respectively (*p* = 0.021). Patients with WR‐DLBCL also had a significantly better 5‐year EFS rate than those with LN‐DLBCL (*p* = 0.021). (B) The 5‐year OS rates of patients with WR‐DLBCL, LN‐DLBCL, and SN‐DLBCL were 83.7%, 70.8%, and 55.8%, respectively (*p* = 0.032). Patients with WR‐DLBCL also had a significantly better 5‐year OS rate than those with LN‐DLBCL (*p* = 0.023).

Patients with WR‐DLBCL, LN‐DLBCL, and SN‐DLBCL had 5‐year EFS rates of 80.7%, 59.5%, and 41.9%, respectively (*p* = 0.021) (Figure 2A) and 5‐year OS rates of 83.7%, 70.8%, and 55.8%, respectively (*p* = 0.032) (Figure 2B). When only WR‐DLBCL and LN‐DLBCL were compared, patients with WR‐DLBCL had a significantly better 5‐year EFS rate (*p* = 0.021) and OS rate (*p* = 0.023) than those with LN‐DLBCL.

**FIGURE 3:**
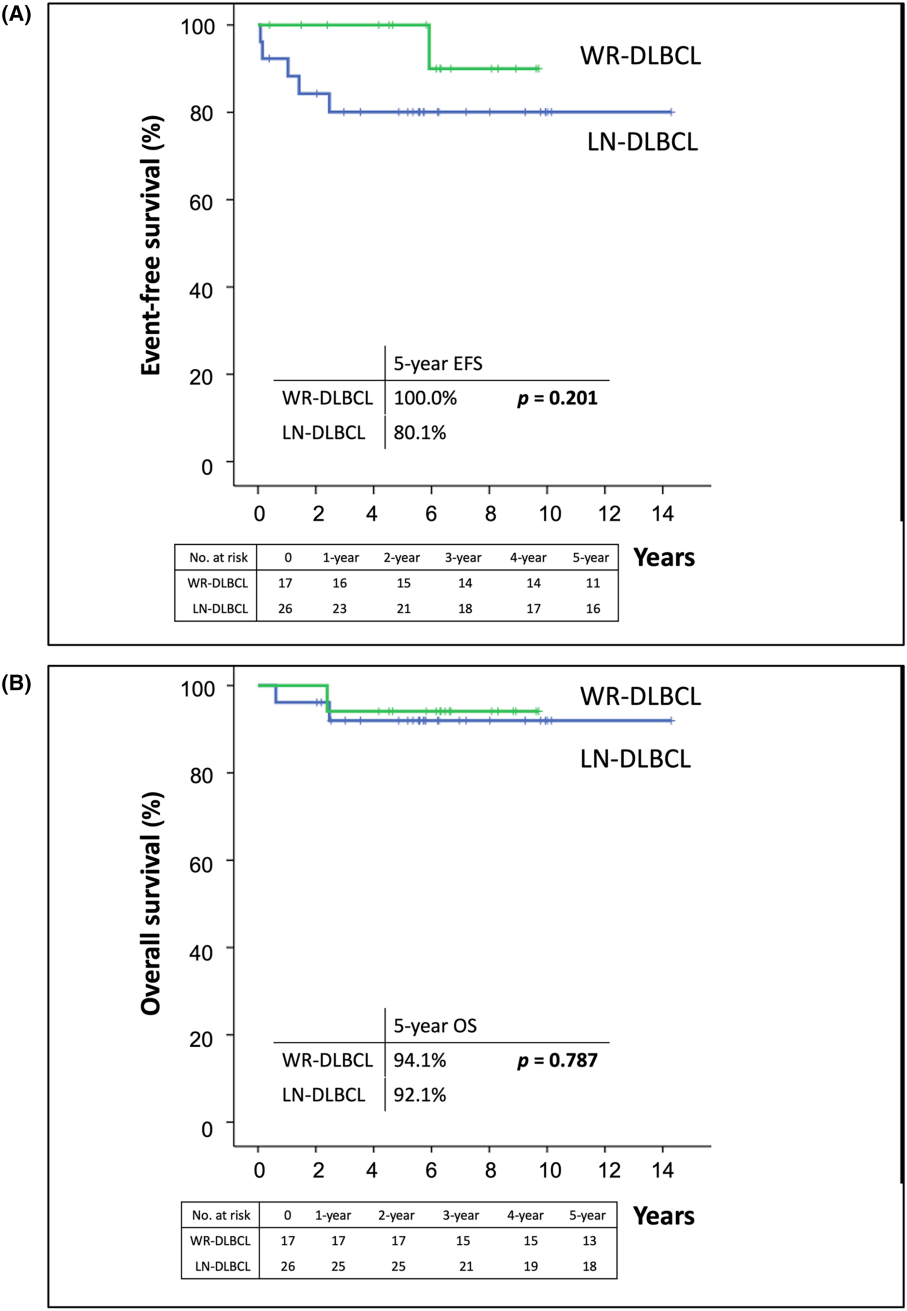
Differences in the event‐free survival (EFS) and overall survival (OS) of patients with diffuse large B‐cell lymphoma involving the Waldeyer ring (WR‐DLBCL) and DLBCL involving lymph nodes without extranodal involvement (LN‐DLBCL), according to revised‐International Prognostic Index (R‐IPI) scores 0. (A) The 5‐year EFS rates of patients with WR‐DLBCL and LN‐DLBCL with R‐IPI scores of 0 were 100.0% and 80.1%, respectively (*p* = 0.201). (B) The 5‐year OS rates of patients with WR‐DLBCL and LN‐DLBCL with R‐IPI scores of 0 were 94.1% and 92.1%, respectively (*p* = 0.787).

**FIGURE 4:**
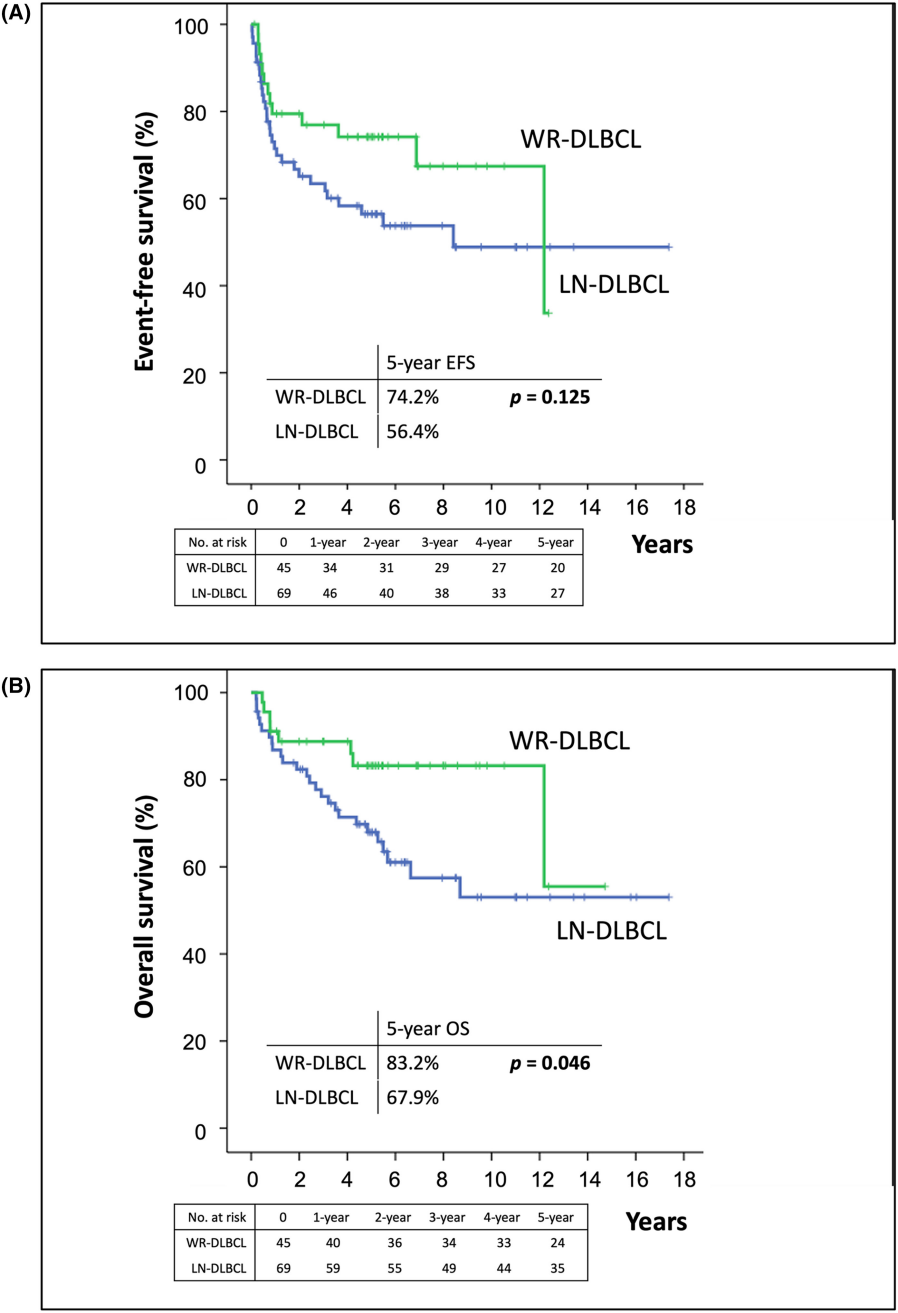
Differences in event‐free survival (EFS) and overall survival (OS) in patients with diffuse large B‐cell lymphoma involving the Waldeyer ring (WR‐DLBCL) and in those with DLBCL involving lymph nodes without extranodal involvement (LN‐DLBCL), according to revised‐International Prognostic Index (R‐IPI) scores 1–2. (A) The 5‐year EFS rates of patients with WR‐DLBCL and LN‐DLBCL with R‐IPI scores of 1–2 were 74.2% and 56.4%, respectively (*p* = 0.125). (B) The 5‐year OS rates of patients with WR‐DLBCL and LN‐DLBCL with R‐IPI scores of 1–2 were 83.2% and 67.9%, respectively (*p* = 0.046).

Regarding R‐IPI score of 0, WR‐DLBCL was associated with a trend toward better 5‐year EFS and OS rates than LN‐DLBCL (EFS rate: 100.0% vs. 80.1%, *p* = 0.201; Figure 3A; OS rate: 94.1% vs. 92.1%, *p* = 0.787, Figure 3B). In addition, with respect to R‐IPI scores of 1–2, WR‐DLBCL was associated with better 5‐year EFS and OS rates than LN‐DLBCL (EFS rate: 74.2% vs. 56.4%, *p* = 0.125, Figure 4A; OS rate: 83.2% vs. 67.9%, *p* = 0.046, Figure 4B). Among patients with R‐IPI score 3, we found the difference in EFS and OS between patients with WR‐DLBCL and LN‐DLBCL was insignificant (EFS, *p* = 0.474; OS, *p* = 0.673; Figure S1).

Among patients with SN‐DLBCL, the prescription of consolidative RT was not associated with 5‐year EFS or OS (RT [*n* = 7] vs. no RT [*n* = 8]; EFS rates: 38.1% vs. 43.8%, *p* = 0.656; OS rates: 44.4% vs. 62.5%, *p* = 0.626). Table 2 provides further information on these 15 patients with SN‐DLBCL. Three patients received front‐line prophylactic intrathecal chemotherapy to prevent DLBCL recurrence in the brain. Two of the three patients had a recurrence in the brain after prophylactic intrathecal chemotherapy, and one of the 13 patients had a recurrence in the brain without previous prophylactic intrathecal chemotherapy. Five patients experienced disease recurrence after front‐line systemic therapy, including two patients with systemically recurrent DLBCL and three patients with brain recurrence. Whole‐brain RT or chemotherapy regimens penetrating the blood–brain barrier were administered to treat three patients with brain recurrence. The patients also received second‐line platinum‐based polychemotherapy to treat systemically recurrent DLBCL (Table 2). In our study, no patients with WR‐DLBCL or LN‐DLBCL received prophylactic intrathecal chemotherapy, and only one patient with WR‐DLBCL (1/67) and one patient with LN‐DLBCL (1/106) had CNS recurrence after receiving frontline treatments.

### 3.3 Prognostic factors for EFS and OS

The prognostic factors for EFS and OS based on univariate and multivariate analyses are listed in Table 3. In the univariate analysis (Table 3), we found that >60 years of age (hazard ratio [HR], 2.113; 95% confidence interval [CI], 1.214–3.679; *p* = 0.008), ECOG performance status ≥2 (HR, 1.932; 95% CI, 1.153–3.235; *p* = 0.012), serum LDH level >1 normal range (HR, 2.499; 95% CI, 1.520–4.109; *p* < 0.001), serum β2‐microglobulin > upper limit of normal range (HR, 2.053; 95% CI, 1.218–3.460; *p* = 0.007), B symptoms (HR, 2.243; 95% CI, 1.310–3.841; *p* = 0.003), R‐IPI scores of 1–2 (HR, 2.920; 95% CI, 1.320–6.460; *p* = 0.008) and 3 (HR, 4.862; 95% CI, 1.876–12.601; *p* = 0.001) were significantly correlated with poor EFS. With respect to DLBCL involvement sites, WR‐DLBCL had a significantly favorable EFS compared to LN‐DLBCL (HR, 0.506; 95% CI, 0.281–0.912; *p* = 0.023). The multivariate analysis showed that R‐IPI scores of 1–2 (HR, 2.858; 95% CI, 1.289–6.336; *p* = 0.010) and 3 (HR, 4.230; 95% Cl, 1.601–11.173; *p* = 0.004) significantly correlated with poor EFS, but WR‐DLBCL (HR, 0.520; 95% CI, 0.288–0.937; *p* = 0.030) significantly correlated with favorable EFS (Table 3).

**TABLE 3:** Univariate and multivariate analyses of prognostic factors to event–free survival and overall survival in diffuse large B‐cell lymphoma involving the Waldeyer ring, sinonasal area, or lymph nodes without extranodal involvement.

Variables	Hazard ratio (95% Cl)	*p* Value	Hazard ratio (95% Cl)	*p* Value
Univariate analysis	EFS		OS	
Age > 60 years	2.113 (1.214–3.679)	**0.008**	3.135 (1.574–6.247)	**0.001**
Sex (male vs. female)	1.551 (0.943–2.551)	0.084	1.572 (0.897–2.757)	0.114
ECOG performance status ≥2 vs. 0–1	1.932 (1.153–3.235)	**0.012**	3.025 (1.722–5.313)	**<0.001**
Serum LDH level >1 normal range	2.499 (1.520–4.109)	**<0.001**	2.620 (1.497–4.586)	**0.001**
Serum β2‐microglobulin > upper limit of normal range	2.053 (1.218–3.460)	**0.007**	1.848 (1.022–3.340)	**0.042**
B symptoms	2.243 (1.310–3.841)	**0.003**	2.996 (1.704–5.266)	**<0.001**
Stage II vs. I	1.830 (0.978–3.425)	0.059	0.992 (0.538–1.828)	0.979
R‐IPI scores				
0	Reference		Reference	
1–2	2.920 (1.320–6.460)	**0.008**	3.906 (1.392–10.957)	**0.010**
3	4.862 (1.876–12.601)	**0.001**	9.792 (3.104–30.894)	**<0.001**
Diffuse large B‐cell lymphoma by involvement sites				
Lymph nodes without extranodal involvement	Reference		Reference	
Waldeyer ring	0.506 (0.281–0.912)	**0.023**	0.465 (0.237–0.913)	**0.026**
Sinonasal area	1.469 (0.690–3.130)	0.318	1.439 (0.605–3.422)	0.410

Multivariate analysis	EFS		OS	
R‐IPI scores				
0	Reference		Reference	
1–2	2.858 (1.289–6.336)	**0.010**	3.793 (1.349–10.664)	**0.011**
3	4.230 (1.601–11.173)	**0.004**	8.475 (2.649–27.110)	**<0.001**
Diffuse large B‐cell lymphoma by involvement sites				
Lymph nodes without extranodal involvement	Reference		Reference	
Waldeyer ring	0.520 (0.288–0.937)	**0.030**	0.508 (0.257–1.001)	0.050
Sinonasal area	1.153 (0.533–2.495)	0.717	0.897 (0.441–2.544)	0.897

Abbreviations: Cl, confidence interval; EFS, event‐free survival; OS, overall survival; R‐IPI scores, revised international prognostic index scores.

*Note*: Bold values indicate significant of *p* value < 0.05.

In the univariate analysis (Table 3), we found that >60 years of age (HR, 3.135; 95% CI, 1.574–6.247; *p* = 0.001), ECOG performance status ≥2 (HR, 3.025; 95% CI, 1.722–5.133; *p* < 0.001), serum LDH level >1 normal range (HR, 2.620; 95% CI, 1.497–4.586; *p* = 0.001), serum β2‐microglobulin > upper limit of normal range (HR, 1.848; 95% CI, 1.022–3.340; *p* = 0.042), B symptoms (HR, 2.996; 95% CI, 1.704–5.266; *p* < 0.001), R‐IPI scores of 1–2 (HR, 3.906; 95% CI, 1.392–10.957; *p* = 0.010) and 3 (HR, 9.792; 95% Cl, 3.104–30.894; *p* < 0.001) significantly correlated with poor OS. According to the DLBCL involvement sites, WR‐DLBCL had a significantly more favorable OS than LN‐DLBCL (HR, 0.465; 95% CI, 0.237–0.913; *p* = 0.026). In the multivariate analysis, R‐IPI scores of 1–2 (HR, 3.793; 95% CI, 1.349–10.664; *p* = 0.011) and 3 (HR, 8.475; 95% Cl, 2.649–27.110; *p* < 0.001) remained poor prognostic factors for OS (Table 3).

### 3.4 IHC staining results for WR‐DLBCL, SN‐DLBCL, and LN‐DLBCL

**FIGURE 5:**
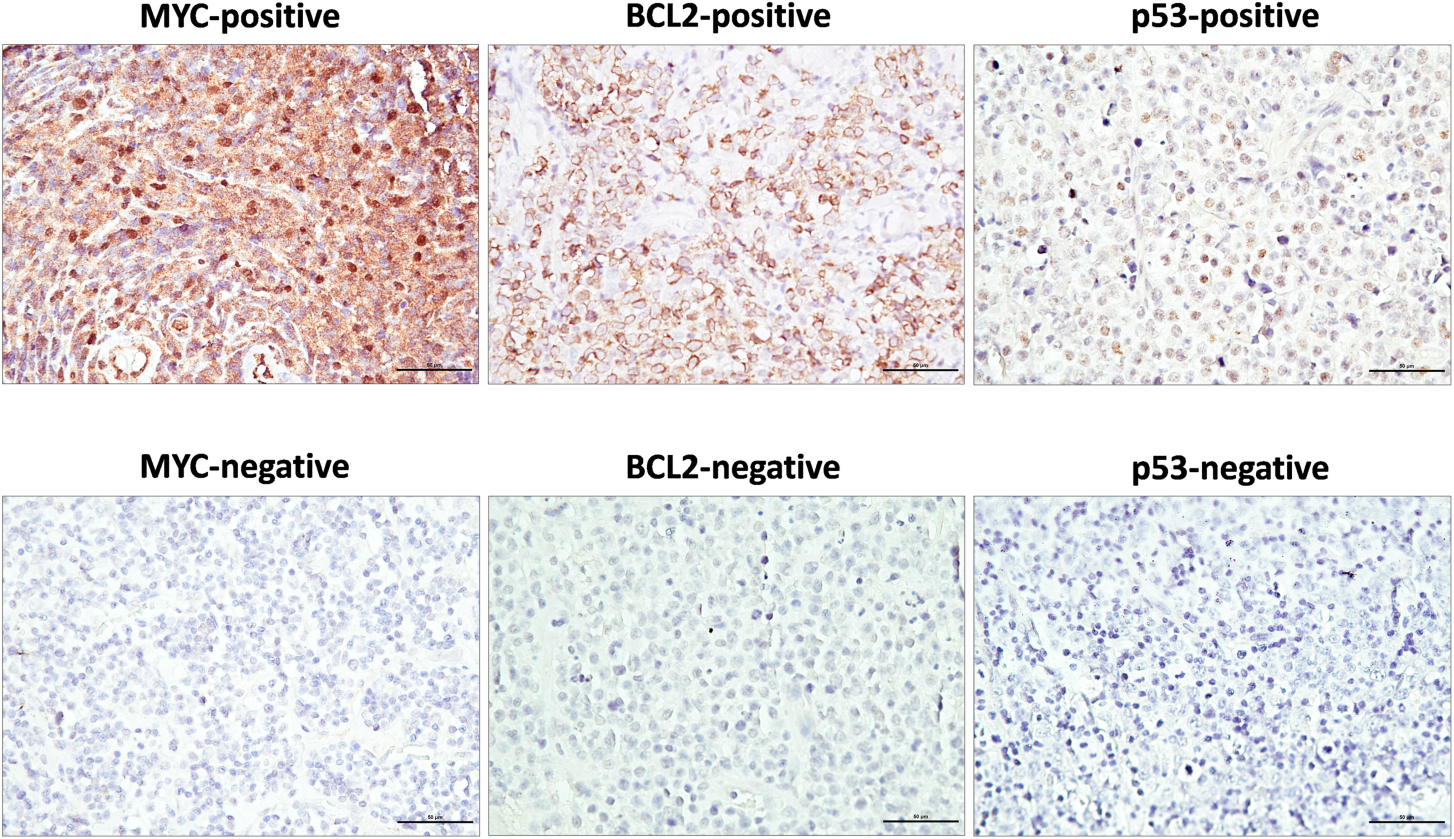
Immunohistochemical staining for MYC, BCL2, and p53 overexpression in diffuse large B‐cell lymphoma involving the Waldeyer ring (WR‐DLBCL), sinonasal area (SN‐DLBCL), or lymph nodes without extranodal involvement (LN‐DLBCL).

In this study, tissue samples from 75 patients were available for further IHC staining and analysis, including 28, 2, and 45 cases of WR‐, SN‐, and LN‐DLBCL, respectively. The positive MYC expression was noted in 5 (18%), 0 (0%), and 7 (16%) of WR‐, SN‐, and LN‐DLBCL, respectively (*p* = 0.795). A total of 11 (39%), 1 (50%), and 21 (47%) patients with WR‐, SN‐, and LN‐DLBCL, respectively, showed positive BCL2 expression (*p* = 0.814). Furthermore, 4 (14%), 0 (0%), and 5 (11%) of WR‐, SN‐, and LN‐DLBCL, respectively, had double expression of MYC and BCL2 (*p* = 0.801). Finally, among patients with WR‐, SN‐, and LN‐DLBCL, 4 (14%), 0 (0%), and 9 (20%) (*p* = 0.662) and 1 (4%), 0 (0%), and 2 (4%) had positive p53 and CD5 protein expression, respectively. Overall, the expression of the aforementioned molecules was not significantly different among the three lymphoma subgroups (Table 4 and Figure 5).

**TABLE 4:** Immunohistochemical staining results of diffuse large B‐cell lymphoma involving the Waldeyer ring, sinonasal area, or lymph nodes without extranodal involvement.

	WR‐DLBCL (*n* = 28)	SN‐DLBCL (*n* = 2)	LN‐DLBCL (*n* = 45)	*p* Value
MYC				0.795
Positive (%)	5 (18)	0 (0)	7 (16)	
Negative (%)	23 (82)	2 (100)	38 (84)	
BCL2				0.814
Positive (%)	11 (39)	1 (50)	21 (47)	
Negative (%)	17 (61)	1 (50)	24 (53)	
Double expression of MYC and BCL2				0.801
Positive (%)	4 (14)	0 (0)	5 (11)	
Negative	24 (86)	2 (100)	40 (89)	
p53				0.662
Positive	4 (14)	0 (0)	9 (20)	
Negative	24 (86)	2 (100)	36 (80)	
CD5				0.942
Positive	1 (4)	0 (0)	2 (4)	
Negative	27 (96)	2 (100)	43 (96)	

Abbreviations: LN‐DLBCL, diffuse large B‐cell lymphoma involving lymph nodes without extranodal involvement; SN‐DLBCL, diffuse large B‐cell lymphoma involving the sinonasal area; WR‐DLBCL, diffuse large B‐cell lymphoma involving the Waldeyer ring.

Regarding the association between expression patterns of MYC, BCL2, double expression of MYC and BCL2, p53, and CD5, and prognosis (EFS and OS) of these subgroups of DLBCLs, we found that the 5‐year EFS rates in patients with WR‐, SN‐, and LN‐DLBCLs were not significantly different regardless of MYC, BCL2, double expression of MYC and BCL2, p53, and CD5 (Table S1). Similarly, we found that the expression patterns of MYC, BCL2, double expression of MYC and BCL2, p53, and CD5 were not associated with OS in patients with WR‐DLBCL, SN‐DLBCL, or LN‐DLBCL (Table S1).

## 4 DISCUSSION

Defining WR‐DLBCL and SN‐DLBC in advanced stages is challenging because DLBCL involves multiple lymphoid organs; thus, we only examined patients with stage I/II DLBCL in the WR and SN areas and compared them with the same period of nodal stage I/II LN‐DLBCL (all patients received rituximab‐based regimen, >80% R‐CHOP regimen). Patients with WR‐DLBCL had significantly more favorable EFS and OS than those with LN‐DLBCL. In addition, patients with SN‐DLBCL exhibited aggressive behavior, including higher R‐IPI scores, higher CNS recurrence rates, and poorer clinical outcomes than those with WR‐DLBCL and LN‐DLBCL. Multivariate analyses showed that R‐IPI scores of 1–2, and R‐IPI score 3 were significantly correlated with worse EFS and OS in localized DLBCL of the head and neck area.

We found that among WR‐DLBCL cases, the palatine tonsil was the most involved site, and SN‐DLBCL represented 8% of head‐and‐neck DLBCLs. Two studies involving Taiwanese populations included 36 and 61 patients with DLBCL located at head and neck area, and the WR area was the most frequently involved site of DLBCL.^27^, ^28^ Previously, López‐Guillermo et al. assessed the differential outcomes of patients with WR‐DLBCL and those with LN‐DLBCL, 90% of whom received CHOP‐based regimens.^12^ López‐Guillermo et al. showed that WR‐DLBCL patients (*n* = 42) had more frequent stage I/II (88% vs. 42%), lower IPI scores (72% vs. 30%), and a better 5‐year OS rate (77% vs. 45%) than those with LN‐DLBCL (*n* = 222).^12^ In another study, Qi et al. evaluated the clinical characteristics and outcomes of 80 patients with WR‐DLBCL and 101 patients with LN‐DLBCL, all were treated with CHOP and 21% received additional rituximab; clinical stage I/II was found in 86% and 71% of WR‐DLBCL and LN‐DLBCL of patients, respectively.^13^ The authors showed that patients diagnosed with WR‐DLBCL and LN‐DLBCL had 5‐year OS rates of 76% and 56%, respectively (*p* = 0.119).^13^ In the GELA (Groupe d'Etude des Lymphomes de l'Adulte) study analyzing 187 patients with WR‐DLBCL (92%, stage I/II disease), all patients received anthracycline‐based polychemotherapy (only 1% patients received rituximab), and the total patients' 5‐year OS rate was 78%.^14^ The IELSG23 study retrospectively collected clinical data from stage I/II head‐and‐neck DLBCL patients.^15^ Among 300 patients with WR‐DLBCL (218 [73%], stage II; 192 [65%], IPI scores of 0–1; >95% received CHOP‐based regimen, >1/2 patients receiving RT), Mian et al. showed a 5‐year OS rate of 74% for this subtype of lymphoma.^15^

In our study, all patients received rituximab‐based chemotherapy, including 88% of the WR‐DLBCL patients and 81% of the LN‐DLBCL patients who received R‐CHOP‐based regimens. Additionally, 13% of the patients with WR‐DLBCL and 19% of the patients with LN‐DLBCL received consolidative RT. Patients with WR‐DLBCL had significantly favorable 5‐year OS rates compared to those with LN‐DLBCL (83.7% vs. 70.8%). Thus, in this study, rituximab‐based regimens improved the outcomes of patients with WR‐DLBCL (in the rituximab era). Other investigators' results showed better OS in patients with localized WR‐DLBCL (in the pre‐rituximab era), indicating that localized WR‐DLBCL is associated with better clinical outcomes (Table 5).

**TABLE 5:** Studies comparing patients' outcomes between diffuse large B‐cell lymphoma involving the Waldeyer ring, sinonasal area, or lymph nodes without extranodal involvement.

Studies	Patient no.	Stages or IPI scores	Treatments	Overall survival
Studies about patients' outcomes between WR‐DLBCL and LN‐DLBCL				
López‐Guillermo A et al.^12^	WR‐DLBCL: 42 LN‐DLBCL: 222	WR‐DLBCL: early stages: 88% low IPI scores: 72% LN‐DLBCL: early stages: 42% low IPI: scores 30%	CHOP regimen: 90%	WR‐DLBCL: 5‐year OS rate: 77% LN‐DLBCL: 5‐year OS rate: 45%
Qi SN et al.^13^	WR‐DLBCL: 80 LN‐DLBCL: 101	WR‐DLBCL: stage I/II: 86% IPI scores 0–1: 75% LN‐DLBCL: stage I/II: 71% IPI scores 0–1: 58%	CHOP or CHOP‐like regimens: 79% R‐CHOP: 21% May receive radiotherapy	WR‐DLBCL: 5‐year OS rate: 76% 5‐year OS rate in stage I/II: 78% LN‐DLBCL: 5‐year OS rate: 56% 5‐year OS rate in stage I/II: 58%
de Leval L et al.^14^	WR‐DLBCL: 187	WR‐DLBCL: stage I/II: 92%	CHOP or CHOP‐like regimens: 99% R‐CHOP: 1%	WR‐DLBCL: 5‐year OS rate: 78%
Mian M et al.^15^	WR‐DLBCL: 300	WR‐DLBCL: stage I/II: 100% IPI scores: 0–1: 65%	CHOP or CHOP‐like regimens: >95% R‐CHOP: rare Radiotherapy: 53% (in total patients with head‐and‐neck DLBCL)	WR‐DLBCL: 5‐year OS rate: 74%
Current study	WR‐DLBCL: 67 LN‐DLBCL: 106	WR‐DLBCL: stage I/II: 100% R‐IPI score 0: 25% R‐IPI scores 1–2: 67% LN‐DLBCL: stage I/II: 100% R‐IPI score 0: 25% R‐IPI scores 1–2: 65%	WR‐DLBCL: Rituximab‐based regimens: 100% R‐CHOP‐based regimens: 88% Radiotherapy: 13% LN‐DLBCL: Rituximab‐based regimens: 100% R‐CHOP‐based regimens: 81% Radiotherapy: 19%	WR‐DLBCL: 5‐year OS rate: 83.7% 5‐year OS rate in R‐IPI 0: 93.3% 5‐year OS rate in R‐IPI 1–2: 83.2% LN‐DLBCL: 5‐year OS rate: 70.8% 5‐year OS rate in R‐IPI 0: 91.3% 5‐year OS rate in R‐IPI 1–2: 61.9%
Studies about patients' outcomes of SN‐DLBCL				
Mian M et al.^15^	DLBCL: 38	SN‐DLBCL: stage I/II: 100% IPI scores 0–1: 70%	CHOP or CHOP‐like regimens: >95%	5‐year OS rate: 75%
Proulx GM et at.^16^	DLBCL: 15 NK cell: 8	Stage I/II: 100%	Radiotherapy: 100% CHOP or CHOP‐like regimens: 13%	5‐year OS rate: 78%
Laskin JJ et al.^17^	DLBCL: 37 T cell: 5 Others: 2	Stage I/II: 100%	CHOP or CHOP‐like regimens: 91% Radiotherapy: 59%	5‐year OS rate: 48%
Peng KA et al.^18^	DLBCL: 9 NK cell: 4 Others: 4		R‐CHOP or R‐ICE regimens: 77% Radiotherapy: 12%	5‐year OS rate: 53%
Current study	SN‐DLBCL: 15	Stage I/II: 100% R‐IPI score 3: 27%	Rituximab‐based regimens: 100% R‐CHOP‐based regimens: 93% Radiotherapy: 47%	5‐year OS rate: 55.8%

Abbreviations: CHOP, cyclophosphamide, doxorubicin, vincristine, and prednisolone; DLBCL, diffuse large B‐cell lymphoma; IPI scores, International Prognostic Index scores; LN‐DLBCL, diffuse large B‐cell lymphoma involving lymph nodes without extranodal involvement; no, numbers; OS, overall survival; R‐CHOP, rituximab, cyclophosphamide, doxorubicin, vincristine, and prednisolone; R‐ICE, rituximab, carboplatin, and ifosfamide; R‐IPI scores, revised International Prognostic Index scores; SN‐DLBCL, diffuse large B‐cell lymphoma involving the sinonasal area; WR‐DLBCL, diffuse large B‐cell lymphoma involving the Waldeyer ring.

In the Southwest Oncology Group (SWOG) S8736 study comparing 3 cycles of CHOP followed by RT with 8 cycles of CHOP in localized intermediate‐ and high‐grade NHL, Miller et al. showed the favorable PFS and OS in patients undergoing consolidative RT.^29^ In the SWOG S0014 study, 3 cycles of R‐CHOP and consolidative RT provided a more favorable PFS than 3 cycles of R‐CHOP alone for patients with limited‐stage, aggressive B‐cell lymphomas.^30^ However, patients in SWOG S8736 and SWOG S0014 studies had continuous relapse during long‐term follow‐up.^30^, ^31^ In the MlnT study, more than 80% of patients had DLBCL, more than 70% were with stage I or II disease, and all patients had with 0–1 IPI scores.^4^ The R‐CHOP‐based regimens of 6 cycles improved patients' EFS and OS when compared to those receiving CHOP‐based regimens.^4^ Overall, these results implied that rituximab‐based immunochemotherapy improved long‐term outcomes of patients who had localized DLBCL. In the current study, all patients received 4–6 cycles of front‐line rituximab‐based regimens, and less than 20% of the patients with WR‐DLBCL and LN‐DLBCL received consolidative RT. Because of adding rituximab, our patients showed improved OS compared with those receiving chemotherapy alone from previous studies. These findings support those of Guan et al.,^32^ which revealed that patients with WR‐DLBCL who received rituximab combined chemotherapy had better PFS and OS than those received chemotherapy.

Owing to the rarity of lymphomas in the SN, most case series have enrolled different lymphoma subtypes for analysis. In the IELSG23 study assessing patients with stage I/II head‐and‐neck DLBCL, 38 patients with stage I/II SN‐DLBCL (>95% receiving CHOP‐based regimens) had a 5‐year OS rate of 75%.^15^ Proulx et al. analyzed 23 patients with stage I/II SN‐lymphomas (15 with DLBCL), all of whom received RT and three also received CHOP‐based regimen, and the 10‐year OS rate for all patients was 78%.^16^ Laskin et al., reported that 44 patients with stage I/II SN lymphomas (37 [84%], DLBCL; 40 [91%] received CHOP or CHOP‐like regimens; >1/2 underwent RT) had a 5‐year‐OS rate of 48%, and the administration of prophylactic intrathecal chemotherapy improved the absolute OS rate by 31% through decreasing CNS recurrence.^17^ Peng et al. studied 17 patients with SN lymphomas, and the most common subtype was DLBCL (nine patients, 53%). Moreover, 13 (77%) patients received rituximab‐based chemotherapy, two (12%) received RT, and the 5‐year OS rate for all patients was 53%.^18^

In this study, patients with stage I/II SN‐DLBCL had higher R‐IPI scores (1–3, 94%) than those with WR‐DLBCL or LN‐DLBCL. This may be because more patients with SN‐DLBCL were older (median age, 73 years; 13 [87%] patients >60 years old) and seven patients had ECOG performance status ≥2. In this study, patients with SN‐DLBCL had worse clinical outcomes than those in the corresponding stages of WR‐DLBCL and LN‐DLBCL, and a high R‐IPI score (score 3) remained an independent poor prognostic factor for all patients, including those with WR‐DLBCL, SN‐DLBCL, and LN‐DLBCL.

Of the 15 patients with SN‐DLBCL, only three received intrathecal prophylactic chemotherapy, of which two (67%) patients and one of the 12 (8%) patients (who did not receive chemotherapy) experienced CNS recurrence. However, all our patients received systemic rituximab, which differed from the methods used by Laskin et al.,^17^ who emphasized the use of intrathecal prophylactic chemotherapy in patients who received the conventional CHOP regimen without rituximab. Thus, in the rituximab era, the beneficial effects of intrathecal chemotherapy for preventing CNS recurrence in patients with SN‐DLBCL should be further explored.

In this study, the frequencies of positive expression of MYC, BCL2, double expression of MYC and BCL2, p53, and CD5 were 16%, 44%, 12%, 17%, and 4%, respectively. The positive rates in our cohort were lower than those from previous studies, with positive expression of MYC, BCL2, double expression of MYC and BCL2, p53, and CD5 in DLBCL at 33%, 53%, 50%, 22%, and 22%, respectively.^24^, ^25^ The possible reasons may be that all our patients had stage I/II disease and most had R‐IPI scores of 0–3, in comparison with previous studies that included patients with stage I to IV disease and IPI scores of 0–5. Teoh et al. reported that patients with stage III/IV DLBCL had a higher frequency of double expression of MYC and BCL2 than those with stage I/II DLBCL (65.7% vs. 34.3%, *p* = 0.381).^33^ Mehta et al. also showed that double expression of MYC and BCL2 was significantly associated with stage III/IV and IPI scores of 3–5 in patients with DLBCL.^34^ Barraclough et al. studied 211 patients with stage I/II DLBCL who received R‐CHOP‐like regimen with or without RT and showed that 33 (16%) had double expression of MYC and BCL2, and the double expression of MYC and BCL2 did not correlate with poor PFS (*p* = 0.552) and OS (*p* = 0.439) in their patients.^35^ Double hits (MYC and BCL2 and/or BCL6 rearrangements) were not associated with poor PFS (*p* = 0.804) or OS (*p* = 0.639) in these patients.^35^ Augustyn et al. reported that among 84 patients with stage I/II DLBCL who received immunochemotherapy followed by consolidative RT, double expression of MYC and BCL2 was detected in 16 (19%) patients; and this double expression was not associated with the inferior PFS (88% vs. 100%, *p* = 0.71) and OS (95% vs. 95%, *p* = 0.40).^36^ These results were in line with analysis of our cohort; 12% tumors harbored double expression of MYC and BCL2, and this double expression did not correlate with the EFS and OS (Table S2). These findings suggest that advanced DLBCL might have a higher double expression of MYC and BCL2, and this double expression may contribute to the poor prognosis of these patients when compared with localized DLBCLs with lower frequencies of double expression of MYC and BCL2.

When we focused on WR‐DLBCL and LN‐DLBCL, we found no significant differences in MYC, BCL2, and double expression of MYC and BCL2, p53, and CD5 in these two subgroups. The CD5 positive rate in DLBCL is low, accounting approximately to 10% of DLBCL, and this subtype of CD5‐positive DLBCL is characterized by aggressive clinical behavior and a higher rate of CNS relapse.^37^, ^38^ However, CD5‐positive DLBCLs have rarely been reported in patients with WR‐DLBCL.^39^ In the current study, we detected CD5 expression in one (4%) of WR‐DLBCL, and this case did not have a relapse after achieving CR, whereas two (4%) of 43 cases of LN‐DLBCL were positive for CD5, and the 5‐year OS for these two cases was 50%. Among the CD5‐positive cases, no CNS relapses were observed. Several studies have revealed that p53 expression, which correlates with *TP53* mutation, is significantly associated with worse OS in patients with DLBCL.^40^, ^41^ However, Chatzitolios et al. showed that OS was not significantly different between patients with and without p53 expression in lymphoma cells of extranodal DLBCL.^42^ In this study, we showed that p53 expression was not associated with OS in patients with WR‐DLBCL. This result is in line with a report by Marques et al., which showed that p53 protein expression did not significantly correlate with EFS and OS in patients with DLBCL involving the palatine tonsils.^43^

Zhang et al. reported that *TP53* mutation accounted for 30% of mutated genes in patients with DLBCL, and 87.5% of *TP53* mutation occurred in exons 5–8 encoding the DNA‐binding domain (DBD) region. They revealed that patients with *TP53* mutation had a trend toward worse median OS than those without *TP53* mutation (92.3 months vs. 110.8 months, *p* = 0.17). In their study, *TP53* mutation was mutually exclusive from *CD58* mutation in patients with DLBCL, and patients' different OS could be stratified by mutation or not in these two genes and their related differential tumor microenvironment.^44^ In the study conducted by Hong, et al.,^45^ the *TP53* mutation was found in 386 (16%) of 2464 patients with DLBCL, and the *TP53* mutation was significantly associated with the worse OS in patients with geminal center B‐cell (GCB) subtype or unclassified subtype but not in activated B‐cell (ABC) subtype. In their study, *TP53* mutation on exons 5–8 encoding the DBD region was significantly associated with worse PFS and OS, whereas *TP53* mutation outside the DBD region was not associated with worse EFS and OS.^45^ In addition, *TP53* mutation on exon 7 was significantly associated with the worse OS, whereas *TP53* mutation on exons 5 and 6 significantly correlated with the poor PFS.^45^ These results suggested that *TP53* mutation on exons 5–7 encoding the DBD region are closely associated with inferior clinical outcome of patients with DLBCL.^44^, ^45^ The lack of data on *TP53* mutation may lead to bias in investigating the prognosis of IHC staining of p53 expression in our patients with WR‐DLBCL, SN‐DLBCL, and LN‐DLBCL. Further analyses of *TP53* mutation in our patients are warranted because this data may provide prognostic values and the differential outcomes of patients with WR‐DLBCL, SN‐DLBCL, and LN‐DLBCL.

Further exploration of novel prognostic factors in the localized stage of WR‐DLBCL is required. As only two cases of SN‐DLBCL with available tissue samples could be analyzed for the expression of MYC, BCL2, p53, and CD5, the enrollment of more patients with SN‐DLBCL, including I to IV, to further assess whether these molecules can serve as prognostic markers in patients with SN‐DLBCL is needed.

In the current study, we revealed that patients with LN‐DLBCL had worse 5‐year EFS and OS rates than those without LN‐DLBCL. Although the differences in expression patterns of molecules such as MYC, BCL2, double expression of MYC and BCL2, p53, and CD5 between WR‐DLBCL subgroups and LN‐DLBCL subgroups were not significant, we showed that patients with LN‐DLBCL had significantly more stage II disease (76% vs. 69%) and bulky disease (7% vs. 1%) than those with WR‐DLBCL. Increased stage II disease and bulky status may contribute to worse clinical outcomes in patients with LN‐DLBCL than in those with WR‐DLBCL. In addition, our study included patients with stages I and II WR‐DLBCL and LN‐DLBCL, which may explain why the frequencies of MYC, BCL2, p53, and CD5 expression were lower in each subgroup than in the literature describing the expression patterns of MYC, BCL2, p53, and CD5 in DLBCL (often stage III/IV). Therefore, it is necessary to explore the underlying biological significance and other prognostic factors in the limited stages of WR‐DLBCL.

The limitations of this study include its retrospective design and diverse chemotherapy dosages, cycles, and imaging follow‐up intervals. These may cause some difference in patients' clinical outcomes in comparison with previous studies. The study data were retrieved between 2000 and 2019. Advancements in clinical care and development of new drugs may result in improved outcomes. Cells of origin, GCB or non‐GCB subtype, and molecular subtypes of DLBCL, such as double‐hit lymphoma (MYC and BCL2 and/or BCL6 rearrangements),^24^, ^35^ diagnosed using interphase dual‐fusion fluorescence in situ hybridization were not included in this study. The lack of sufficient available tissue samples (only 40%) prevents the assessment of the expression pattern of the combined expression of MYC and BCL2. Additionally, the lack of data of double hits does not determine whether biological significance of these molecules contributes to the differential clinical outcomes of limited stages of WR‐DLBCL and LN‐DLBCL. In future studies, we will investigate potential molecular prognostic factors and new treatment strategies in patients with WR‐DLBCL with high R‐IPI scores and in those with SN‐DLBCL.

In conclusion, our current study demonstrated that in the rituximab era, patients with stage I/II WR‐DLBCL had more favorable 5‐year EFS and OS rates than those with stage I/II LN‐DLBCL, indicating that WR‐DLBCL is a disease entity with better outcomes than nodal DLBCL. In our study, a rare subtype of SN‐DLBCL was closely associated with a higher R‐IPI score, more frequent CNS relapses, and poorer prognosis. Additional molecular studies and new treatment strategies are warranted for this DLBCL subtype.

## AUTHOR CONTRIBUTIONS

**Wei‐Li Ma:** Data curation (equal); formal analysis (equal); investigation (equal); writing – original draft (equal). **Ruey‐long Hong:** Investigation (equal); resources (equal). **Pei‐Jen Lou:** Investigation (equal); resources (equal). **Ming Yao:** Investigation (equal); resources (equal). **Shang‐Ju Wu:** Investigation (equal); resources (equal). **Chung‐Wu Lin:** Investigation (equal); methodology (equal). **Chun‐Wei Wang:** Investigation (equal); resources (equal). **Chin‐Hao Chang:** Formal analysis (equal); methodology (equal). **Ann‐Lii Cheng:** Supervision (equal). **Sung‐Hsin Kuo:** Project administration (equal); supervision (equal); writing – original draft (equal); writing – review and editing (equal).

## FUNDING INFORMATION

The National Science and Technology Council supported research grants No. 110‐2314‐B‐002‐219‐MY3, No. 111‐2314‐B‐002‐010‐, No. 111‐2811‐B‐002‐095‐, and No. 112‐2314‐B‐002‐156‐; and the National Taiwan University Hospital supported grant No. 112‐S0215 for this study.

## CONFLICT OF INTEREST STATEMENT

All authors of this study have no conflicts of interest to declare.

## Supporting information


Figure S1.

Table S1.

Table S2.

## Data Availability

The datasets used and analyzed within the present study are available from the corresponding author on reasonable request.
