# Supplementary figures and images for: Improved outcomes of localized diffuse large B‐cell lymphoma at the Waldeyer ring in comparison to the sinonasal area in the rituximab era
Source: Cancer Med. 2023 Dec 26;13(1):e6851. doi: 10.1002/cam4.6851 (PMC10807621; doi:10.1002/cam4.6851)

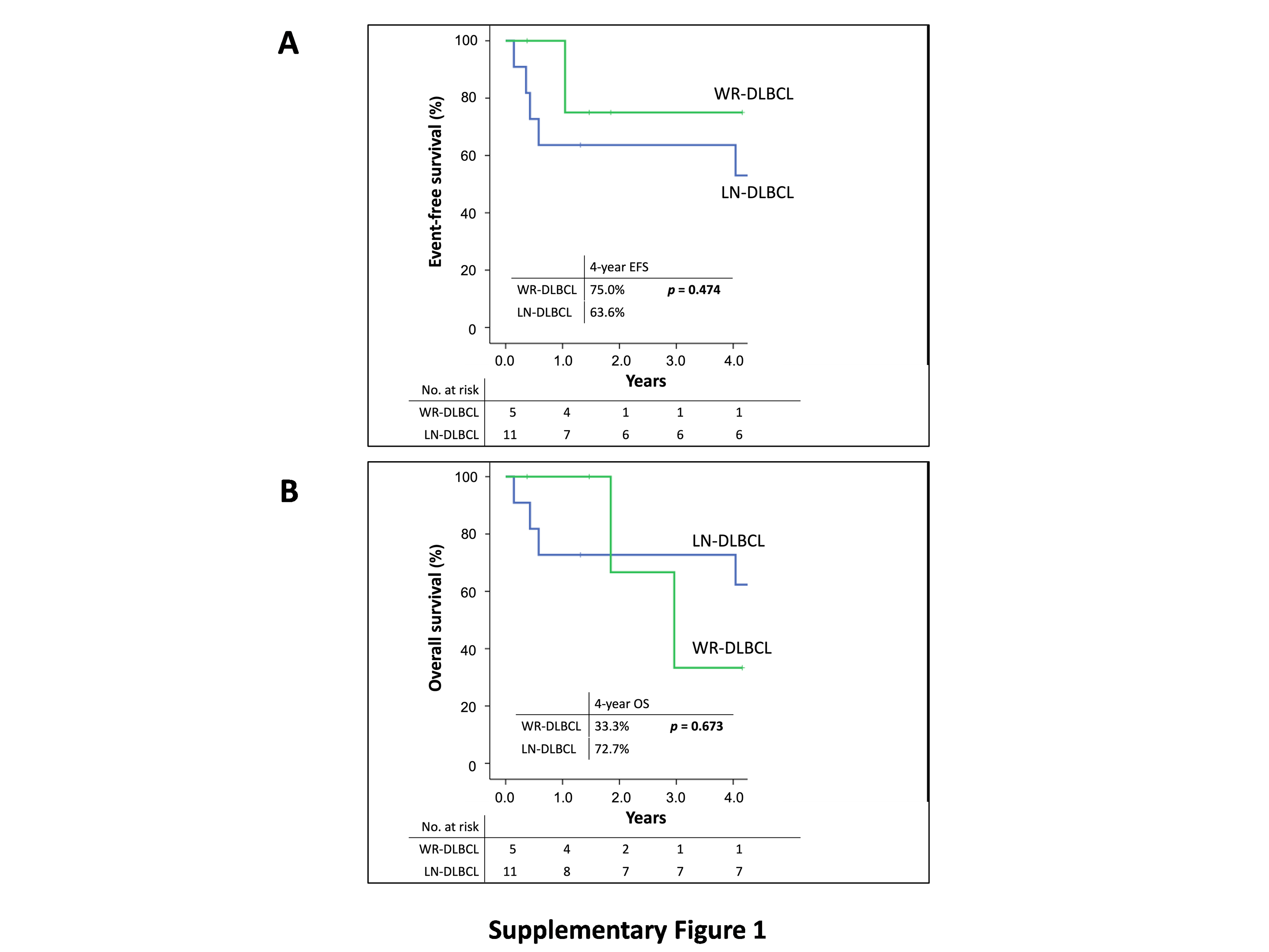

Supplement: Supplementary file 1 — Figure S1. [file CAM4-13-e6851-s002.tif]
