# Supplementary material for: Improved outcomes of localized diffuse large B‐cell lymphoma at the Waldeyer ring in comparison to the sinonasal area in the rituximab era
Source: Cancer Med. 2023 Dec 26;13(1):e6851. doi: 10.1002/cam4.6851 (PMC10807621; doi:10.1002/cam4.6851)
Supplement: Supplementary file 2 — Table S1. [file CAM4-13-e6851-s003.docx]

**Supplementary Table 1. Five-year event-free survival and overall survival rate of diffuse large B-cell lymphoma involving the Waldeyer ring, sinonasal area, or lymph nodes without extranodal involvement according to MYC, BCL2, p53 and CD5 expression**

|  | **WR-DLBCL (n = 28)** | | | | **SN-DLBCL (n = 2)** | | | | **LN-DLBCL (n = 45)** | | | |
| --- | --- | --- | --- | --- | --- | --- | --- | --- | --- | --- | --- | --- |
|  | **5-y EFS rate** | **p value** | **5-y OS rate** | **p**  **value** | **5-y EFS rate** | ***p* value** | **5-y OS rate** | **p value** | **5-y EFS rate** | **p value** | **5-y OS rate** | **p**  **value** |
| MYC |  |  |  |  |  |  |  |  |  |  |  |  |
| Positive (%) | 100% | 0.208 | 100% | 0.456 | nil |  | nil |  | 21.4% | 0.069 | 68.6% | 0.761 |
| Negative (%) | 77.1% |  | 86.7% |  | 50.0% |  | 50.0% |  | 63.7% |  | 74.2% |  |
| BCL2 |  |  |  |  |  | 0.317 |  | 0.317 |  |  |  |  |
| Positive (%) | 100% | 0.044 | 100% | 0.132 | 0 |  | 0 |  | 70.6% | 0.287 | 85.7% | 0.145 |
| Negative (%) | 66.2% |  | 80.8% |  | 100% |  | 100% |  | 45.5% |  | 61.8% |  |
| Double expression of  MYC and BCL2 |  |  |  |  |  |  |  |  |  |  |  |  |
| Yes (%) | 100% | 0.223 | 100% | 0.456 | nil |  | nil |  | 60.2% | 0.309 | 100% | 0.115 |
| No (%) | 77.4% |  | 86.7% |  | 50.0% |  | 50.0% |  | 40.0% |  | 70.1% |  |
| p53 |  |  |  |  |  |  |  |  |  |  |  |  |
| Positive (%) | 100% | 0.318 | 100% | 0.456 | nil |  | nil |  | 77.8% | 0.222 | 100% | 0.155 |
| Negative (%) | 77.4% |  | 86.7% |  | 50.0% |  | 50.0% |  | 52.4% |  | 67.2% |  |
| CD5 |  |  |  |  |  |  |  |  |  |  |  |  |
| Positive (%) | 100% | 0.642 | 100% | 0.727 | Nil |  | nil |  | 50.0% | 0.880 | 50.0% | 0.737 |
| Negative (%) | 80.2% |  | 88.3% |  | 50.0% |  | 50.0% |  | 58.0% |  | 74.5% |  |

Abbreviations: WR-DLBCL, diffuse large B-cell lymphoma involving the Waldeyer ring; SN-DLBCL, diffuse large B-cell lymphoma involving the sinonasal area; LN-DLBCL, diffuse large B-cell lymphoma involving lymph nodes without extranodal involvement; 5-y EFS, 5-year event-free survival; 5-y OS, 5-year overall survival
