# Supplementary material for: Improved outcomes of localized diffuse large B‐cell lymphoma at the Waldeyer ring in comparison to the sinonasal area in the rituximab era
Source: Cancer Med. 2023 Dec 26;13(1):e6851. doi: 10.1002/cam4.6851 (PMC10807621; doi:10.1002/cam4.6851)
Supplement: Supplementary file 3 — Table S2. [file CAM4-13-e6851-s001.docx]

**Supplementary Table 2. Five-year event-free survival and overall survival rate of diffuse large B-cell lymphoma according to MYC, BCL2, p53 and CD5 expression**

|  | **5-y EFS rate** | **p value** | **5-y OS rate** | **p value** |
| --- | --- | --- | --- | --- |
| MYC |  |  |  |  |
| Positive (%) | 51.9% | 0.517 | 80.2% | 0.484 |
| Negative (%) | 67.8% |  | 77.7% |  |
| BCL2 |  |  |  |  |
| Positive (%) | 78.5% | 0.077 | 87.9% | 0.151 |
| Negative (%) | 53.5% |  | 69.3% |  |
| Double expression of MYC and BCL2 |  |  |  |  |
| Yes (%) | 66.7% | 0.904 | 100% | 0.068 |
| No (%) | 65.4% |  | 75.0% |  |
| p53 |  |  |  |  |
| Positive (%) | 84.6% | 0.135 | 100% | 0.054 |
| Negative (%) | 61.0% |  | 73.3% |  |
| CD5 |  |  |  |  |
| Positive (%) | 66.7% | 0.854 | 66.7% | 0.813 |
| Negative (%) | 65.4% |  | 78.5% |  |

Abbreviations: 5-y EFS, 5-year event-free survival; 5-y OS, 5-year overall survival
